# International and Spanish Findings in Scientific Literature about Minors’ Mental Health: Predictive Factors Using the Strengths and Difficulties Questionnaire

**DOI:** 10.3390/ijerph16091603

**Published:** 2019-05-08

**Authors:** Fernando Fajardo-Bullón, Irina Rasskin-Gutman, Benito León-del Barco, Eduardo João Ribeiro dos Santos, Damián Iglesias Gallego

**Affiliations:** 1 Department of Psychology, Faculty of Education and Psychology, University of Extremadura, 06006 Badajoz, Spain; 2 Department of Psychology, Teacher Training College, University of Extremadura, 10003 Cáceres, Spain; irasskin@unex.es (I.R.-G.); bleon@unex.es (B.L.-d.B.); 3 Scientific Coordinator R&D Unit Institute of Cognitive Psychology (IPCDHS/FPCE), University of Coimbra, 3000-115 Coimbra, Portugal; eduardosantos@fpce.uc.pt; 4 Department of Didactics of Music, Plastic and Body Expression, Teacher Training College, University of Extremadura, 10003 Cáceres, Spain

**Keywords:** mental health, gender, age, social class, child welfare

## Abstract

Minors’ mental health is a subject of high global concern. Understanding the factors that influence their mental health is essential to improving the health of future generations. In this study, an analysis of the Strengths and Difficulties Questionnaire’s usefulness is carried out, as a validated tool, recognized in Spain and internationally, for the measurement of minors’ mental health. In turn, the influence of the variables of gender, age, and physical health, along with the occupational social class of parents on Spanish minors’ mental health, has been analyzed. Spanish minors with good physical health and of parents with middle and higher education, as well as in an occupational social class, are less likely to suffer mental health problems. On the other hand, it seems that internalizing symptoms are more likely in girls, and externalizing symptoms are more likely in boys. However, when a global measure of mental health is made without specific subscales, the effects of gender and age diverge greatly, according to the studies. Although there are examples of current research using the same measurement tool, there is still a need for many more international studies that are coordinated using the same methodology. This study identifies the factors which the international and Spanish scientific literature has revealed as being determinants in minors’ mental health. Finally, it is essential that the influence of these factors be assessed in the areas of primary care and mental health to facilitate better detection, intervention, or prevention of mental health problems in today’s children, as well as the children of future generations.

## 1. Introduction

The analysis of minors’ mental health has become a topic of research of global interest, with recent international contributions from all continents: Africa [1], Europe [2], Asia [3], Oceania [4], and America [5]. Overall, it is estimated that between 10% and 20% of minors suffer from mental health problems [6]. A recent meta-analysis of 41 studies in 27 countries, with a total of 87,742 children, established a worldwide prevalence of 13.4% in children and adolescents, with 12.36% in children aged specifically between 6 and 11 years [7].

On the other hand, it has been shown that half of adults’ disorders have their beginning in adolescence [8]. Despite this, more than half of the children with mental problems do not receive specialized treatment in mental health [9]. In turn, some international studies have shown a greater proportional increase in mental health disorder diagnoses in the juvenile population compared to adults although, in both cases, there was an augmentation in the use of psychiatric medication [10]. This increase in the number of mental health problems in children [6], especially in Western countries [11,12], implies a clear need to analyze mental health in the early stages, not only to improve intervention, but also as a preventative approach.

Regarding the explanatory variables of mental health in children and adolescents, several studies within the Spanish context have focused their attention on analysis of the influence of variables such as age, gender, occupational social class of parents, or perceived physical health [13,14,15,16]. The early identification of these variables can be beneficial in helping to avoid emotional and behavioral problems that can also be aggravated at later stages of life. This paper presents an analysis of mental health associated with these variables in the Spanish population compared to the international scientific literature. In turn, an analysis of the usefulness of the Strengths and Difficulties Questionnaire (SDQ), to measure the mental health of the population between 4 and 16 years old, is presented.

## 2. Materials and Methods

### 2.1. Evaluation of Minors: Use of the Strengths and Difficulties Questionnaire

Since the second half of the twentieth century, attempts have been made to measure the population health of some countries through national health surveys [17]. Unfortunately, in the beginning, the diversity of methodologies and instruments made it difficult to compare results internationally [7]. However, thanks to the introduction of internationally validated questionnaires into the scientific community, samples can be compared and more global analyses carried out [2]. In this sense, the international agreement for the application of the Strengths and Difficulties Questionnaire (SDQ) [18], or the measurement of mental health, has represented an advance in research coordination within the scientific community [19], always with the precaution of the possible effects that cultural differences may have on the results [20,21]. This questionnaire has been validated internationally [4,15,22,23,24,25] and has been used in a multitude of studies to measure mental health both longitudinally [4,26], and transversally [14,27]. It has proven to be an excellent screening tool compared to other more classic tools, such as the Child Behavior Checklist (CBCL) [28,29]. At the same time, its ease of use and internal structure make it very attractive for research [22,30]. It has demonstrated good use in detecting probable cases of psychological problems in European children, with good psychometric properties across seven European countries [31]. A systematic review by Kersten et al. showed interesting comparative information of the psychometric properties obtained in several studies, using the teacher and parent version of the SDQ [32]. It consists of five subscales of five items each. The total score for difficulties or mental health problems is calculated using the sum of the subscales of conduct problems, emotional problems, peer relationship problems, and hyperactivity [22], ranging from 0 to 40 points. The fifth subscale, prosocial behavior, is not considered for the valuation of the total score. Regarding the psychometric properties of the SDQ, one recent systematic review by Ortuño-Sierra et al. [19] shows that most studies prove the existence of five solid and well-defined factors that correspond to the SDQ subscales. This review describes more than 50 international studies, carried out between 2000 and 2015, that analyze the validity of the questionnaire construct by means of exploratory factor analysis (EFA) and confirmatory factor analysis (CFA). On the one hand, the values that achieve goodness of fit in the five-factor model (emotional symptoms, conduct problems, peer relationship problems, hyperactivity, and prosocial behavior) are found; although, some studies found goodness of fit in the two-factor second-order model [33]. On the other hand, regarding the reliability of the SDQ, it can be pointed out that the total score, as well as the subscales of emotional symptoms, conduct problems, peer relationship problems, hyperactivity, and prosocial behavior, show good internal consistency, whereas the conduct problems and peer relationship problem subscales present more discrete Cronbach’s alpha values [19]. This questionnaire also allows the use of two factors, internalizing (sum of the emotional problems and peer relationship problems subscales) and externalizing factors (conduct problems and hyperactivity) [34], and could be a good screening tool for attention-deficit/hyperactivity disorder (ADHD) [35]. However, recent studies with the SDQ-parent version in the Spanish population highlight that a structure of five factors is the best choice, avoiding bifactorial use [34]. However, the different structures found could explain the manifestation of psychological difficulties in children and adolescent populations. The questionnaire allows the use of different versions depending on the sample, self-report for adolescents between 11 and 16 years old, and the versions for parents and teachers, and for children from 4 to 16 years old. The self-report version is recommended for people over 11 years old [36] although good psychometric qualities have also been obtained when it was used with children between 8 and 13 years old [37]. Even so, the parent version is the most commonly used for measuring the mental health of Spanish children [38]. In Spain, it was introduced for the first time in the National Health Survey of 2006 to study the mental health of children. In addition to the 2006 Spanish National Health Survey, ENSE 2006 [13,15,38,39], it has also been used in subsequent counterpart studies, ENSE 2011-2012 [14,34]. Therefore, it can be concluded that the SDQ-parent is an internationally recognized instrument with excellent quality for the screening of children with problems or difficulties in mental health [40]. The most recent studies recommend the use of more than one version at the same time, for example, using both the parental and self-reported versions [36]. Finally, there is an extended version of the SDQ with an impact supplement, to better explore the child’s or adolescent’s social difficulties. It explores four domains: friendships, classroom learning, home life, and leisure activities (not for teacher or caregiver) [41]. New questions for use after interventions are introduced in the follow-up versions of the SDQ (https://www.sdqinfo.org).

### 2.2. Influence of Gender and Age on Mental Health

Regarding the influence of the variable of gender on mental health during childhood and adolescence in Spain, we find that in most of the studies reviewed, independently of which SDQ version was used, i.e., the parent’s version or self-reported version, females tend to score higher in internalized symptoms, such as emotional problems and also at the prosocial behavior subscale, whereas males score higher in external symptoms, such as behavioral problems and hyperactivity [14,34,38,42]. These results converge with the outcomes from a large volume of studies obtained with samples from other countries [25,40,43,44,45,46,47,48,49,50].

However, it has also been observed, in some Spanish studies, that there were no apparent significant differences in terms of gender [15,51] or, specifically, in relation to the dimension of peer relationship problems [38]. In another study using an international sample [40], no significant gender differences were observed regarding internalizing disorders, though a greater prevalence was found in adolescent females compared to males. In this study, males hyperactivity/inattention and conduct disorder were also consistently associated with a higher probability of disorders [52]. These differences could be related to a lack of clear diagnosis criteria, as well as the actual diversity of evaluation methodologies for measuring pathological symptoms in boys and girls [13,15].

Regarding the variable age, no conclusive general findings were shown in the reviewed Spanish studies. This is consistent with other previous works [14,53]. Taking into account specific measures on the different SDQ dimensions, some of the analyzed studies point out that females emotional problems scores increase with age, and scores in hyperactivity and conduct problems were lower in the older age group in the total sample, whereas scores in prosocial behaviors were higher in the older age group for the total sample [38]. However, when comparing age groups, other studies reveal that the elder group (14–18 years) presents higher scores in emotional, conduct, and hyperactivity problems, as well as the Total Difficulties Score, than the younger group [54]. In this sense, some international studies found a growing tendency in symptomatology as subjects grew up [25,44,50], whereas others found a reverse tendency [13,45,48] or no association [11,55].

### 2.3. Physical State of the Minor and Mental Health

The association between perceived physical health and mental health in children and adolescents has also been studied in the Spanish population through the SDQ [15]. Several international studies have shown how healthy lifestyles are associated with a lower risk of suffering from mental disorders [56]. In this regard, it is worth mentioning the importance of regular physical activity as a precursor to good physical and mental health [57]. Thus, it has been found that levels of obesity and physical activity practice can predict mental health states in Spanish children and adolescents [58]. In this way, participation in extracurricular sports activities has also been shown as a variable associated with good mental health, especially in the case of boys [59]. From another point of view, recent Spanish studies show how perceived poor physical health may favor the possibility of suffering mental health problems [13,15,54]. These results have also been supported in other international investigations demonstrating that poor physical health is a risk factor for mental health [60,61].

### 2.4. Occupational Social Class and Educational Level of Parents Associated with Minor’s Mental Health

Mentioned below are the main Spanish studies on family variables and mental health measured through the SDQ. This analysis summarizes the results according to various factors, such as the level of academic studies and occupational social class. The results of these Spanish studies show a strong association between parent education and child mental health as informed by the parents [62,63]. Likewise, the children of mothers with a primary education level have worse mental health than the children of mothers with university studies [64], with a high level of education being considered a factor that reduces children’s mental health problems [65]. These results, obtained from the general population of Spanish minors, agree with international research that demonstrates an association between the low level of training and socioeconomic status of parents, and with a minor’s poor mental health [66,67,68]. This association is still present in the last European Health Survey in Spain (EHSS 2014) and, more globally, in the most recent European Health Interview Survey (EHIS) [69].

Regarding the occupational social class, this category has been classified in Spain according to the proposal of the Spanish Society of Epidemiology’s Working Group (SEE) [70], or according to the Spanish adaptation of the British General Registrar’s classification [13]. For a simpler comparative analysis, the occupational class is usually divided into three types: high, medium, and low class. The results show how the jobs of the main breadwinner in lower categories are more strongly related to the presence of mental health problems in children [63,71]. On the contrary, it is the families with jobs corresponding to the middle classes and the most privileged classes that suppose a protective factor against the more disadvantaged classes concerning the presence of mental health problems in minors [14,62,63,64]. These data, obtained from the general population of Spanish minors, agree with other international research that shows that children from families with a poorer socioeconomic status have worse physical and mental health than minors with a high socioeconomic level [68,72,73,74].

### 2.5. Family Variables and Mental Health of Minor’s Family Variables and Minor’s Mental Health

Family variables have been shown to be of great importance in the minor’s mental health [13,16]. The parent’s mental health [60] and socioeconomic status [39], or the children’s healthcare [15], are some of the variables that have proven to be determining factors. Other variables that have been shown to be significant for a child’s inadequate mental health have been the type of single-parent family [37,62,63], as well as the presence of other relatives in situations of unemployment [65]. Likewise, the influence of poor mental health of the parents [63] and, specifically, of the mother [65], has been analyzed as a determining variable for the development of the mental well-being of their children. These results coincide with other international research that shows how parent’s inadequate mental health negatively affects the health of their children [60,75]. With respect to Spain, the main research on family variables and mental health, as measured with the SDQ, has used the total or partial sample and data from the National Health Survey ENSE 2006 and/or ENSE 2012. This has had great advantages regarding ensuring of the validity of sources or external evidence, and the generalization of the results in Spain but, in turn, has been limited regarding the objectives of the research. The methodology and sample size of these surveys, representative of the entire Spanish population, are a great advantage. However, only the parent version of the SDQ has been used, and the level of studies, social class, and parental health have been analyzed mainly as family variables. In this sense, it is advisable to advance the research using other versions of the SDQ in Spain, such as the self-report with children from the age of 11. Likewise, there are other variables, such as family relationships, from the point of view of family violence, either between parents [76], or from parents to children [77], that should also be analyzed. In this sense, the theory of parental rejection acceptance [78] could be interesting for analyzing the influence of parental affections on their children, and how these perceptions or experiences can affect the cognitive and socioemotional adjustment of minors [79]. It is known that lack of affection on the part of the mother, together with rejection from the father, can be related to a greater probability of aggressive behaviors in situations of bullying [80], causing, in turn, the minor’s mental health problems involved in these behaviors [81]. Even so, more studies are needed to analyze the filioparental relationships of affection/rejection and its relationship with the minor’s mental health.

## 3. Conclusions

The global situation of minors’ mental health is alarming. The high figures, where 2 out of 8 children suffer from these pathologies, indicate the relevance of this theme. In this study, the SDQ questionnaire is shown to be a recognized and recommended international tool for the measurement of mental health problems of minors both in Spain and internationally. In turn, a middle and high social class and educational level of parents has been shown to be a preventive factor of mental health problems of minors both in Spain and internationally. Similarly, good physical health of the child is shown as a factor associated with good mental health in both Spanish and international studies. Regarding gender and age, there are studies that associate externalizing symptoms to the masculine gender and internalizing to the feminine, although there is still a need for more international studies that clarify the diversity of currently published results. In turn, complementary studies that analyze, in greater depth, the family affective relationships between parents and children as an influential factor in mental health seem necessary. Similarly, an analysis of the variables associated with sedentary lifestyle, such as online gaming, should be researched in a greater number of studies, in order to understand their possible influence on the relationship between physical health and mental health in minors, using the SDQ. Finally, in accordance with other researchers [19], we also consider it reasonable that advances in the detection of factors associated with the mental health of minors will have repercussions in different areas—medical, psychological, and academic—in addition to influencing public policies, due to the active pursuit of these advances by patients and relatives.
